# The Prognostic Value of the Endothelial Activation and Stress Index in Stable Patients with Idiopathic Pulmonary Arterial Hypertension

**DOI:** 10.3390/jcm15208201

**Published:** 2026-10-21

**Authors:** Kurtulus Karauzum, Irem Yilmaz, Aysen Simsek Kandemir, Hooman Rahimipour

**Affiliations:** 1 Cardiology Department, Kocaeli University Hospital, 41380 Kocaeli, Türkiye; karauzumirem@gmail.com (I.Y.); hrahimmi.66@gmail.com (H.R.); 2 Biostatistics Department, Kocaeli University, 41200 Kocaeli, Türkiye; aysens@kocaeli.edu.tr

**Keywords:** pulmonary arterial hypertension, endothelial activation and stress index, endothelial dysfunction, inflammation, hemodynamics, mortality, risk stratification

## Abstract

**Background**: Endothelial dysfunction and chronic inflammation are central pathophysiological mechanisms in the development of idiopathic pulmonary arterial hypertension (IPAH). EASIX, the endothelial activation and stress index, is a simple and recently introduced score that captures endothelial activation and systemic stress. EASIX has been shown to be associated with several cardiovascular conditions. We investigated the relationship between EASIX and clinical outcomes in stable patients with IPAH. **Methods**: We retrospectively analyzed a total of 256 stable patients with IPAH in this single-center prevalent cohort. EASIX was calculated from lactate dehydrogenase, creatinine, and platelet count. The optimal cut-off of EASIX was derived by receiver operating characteristic (ROC) analysis in this cohort without external validation. Its association with all-cause mortality was assessed with ROC analysis, Kaplan–Meier estimates, and Cox regression models adjusted for age, sex, World Health Organization (WHO) functional class (WHO-FC), 6-min walking distance (6-MWD), and N-terminal pro B-type natriuretic peptide (NT-proBNP), and its incremental value with Harrell’s C-index. **Results**: Forty-one patients (16.0%) died during a median follow-up of 35 (interquartile range 21–46) months. In multivariable Cox regression analysis, EASIX was independently associated with all-cause mortality both as a continuous variable (Model 1; hazard ratio (HR) per 1-unit increase 1.528, 95% confidence interval (CI) 1.255–1.860; *p* < 0.001) and when dichotomized at ≥1.23 (Model 2; HR 3.989, 95% CI 1.962–8.111; *p* < 0.001). Adding EASIX to a model with WHO-FC, 6-MWD, and NT-proBNP changed Harrell’s C-index from 0.784 to 0.812 (likelihood-ratio test *p* < 0.001). The patients with higher EASIX had worse functional capacity, right ventricular function, and hemodynamics. WHO-FC III–IV and logNT-proBNP were also independently associated with all-cause mortality. **Conclusions**: In this single-center cohort of patients with IPAH, higher EASIX was associated with more advanced disease and was independently associated with long-term all-cause mortality. Its incremental value over established risk-stratification tools and the reproducibility of the data-derived cut-off require external validation.

## 1. Introduction

Idiopathic pulmonary arterial hypertension (IPAH) is a rare disease that worsens over time and can be fatal. Remodeling of the small pulmonary arteries raises pulmonary vascular resistance and eventually impairs right heart function [1]. Targeted drug therapies have improved considerably over the past two decades, yet IPAH is still associated with considerable morbidity and mortality [2]. Therefore, early identification of patients at increased risk of adverse outcomes is crucial to guide treatment decisions and improve long-term outcomes.

Contemporary prognostic evaluation in IPAH relies on multiparametric risk stratification rather than on a single biomarker. Current international guidelines recommend a comprehensive three-strata model at diagnosis and a simplified four-strata model during follow-up, integrating clinical status, exercise capacity, natriuretic peptides and, when appropriate, imaging, and hemodynamic variables [3]. Other validated approaches include the COMPERA 2.0 four-strata model and the REVEAL Lite 2 risk score [4]. These established multidimensional tools therefore represent the clinical benchmark against which the potential value of novel prognostic markers should ultimately be considered.

In IPAH, endothelial dysfunction contributes to both the onset and the progression of disease [5]. The damaged endothelium shifts the balance of vasoactive mediators toward vasoconstriction, amplifies inflammatory signaling, and drives the growth of pulmonary artery smooth muscle cells, so the vessel wall is remodeled step by step. These alterations progressively increase right ventricular afterload, leading to right ventricular failure [5,6]. Given the pivotal role of endothelial dysfunction in IPAH, biomarkers reflecting endothelial inflammation may also provide valuable prognostic information beyond traditional clinical and hemodynamic parameters [7]. The endothelial activation and stress index (EASIX), calculated from serum lactate dehydrogenase (LDH), serum creatinine, and platelet count, has recently emerged as a simple and readily available surrogate marker of endothelial activation and systemic stress [8]. Importantly, each of its individual components has previously been associated with prognosis in PAH. Higher serum LDH levels have been associated with greater disease severity and mortality in IPAH; increased serum creatinine has been associated with adverse hemodynamics and mortality in PAH; and thrombocytopenia has been reported as an independent predictor of mortality in IPAH [9,10,11]. Thus, the individual components of EASIX already carry prognostic information in PAH, while whether their combination within EASIX provides a useful composite measure in IPAH remains uncertain. EASIX was first described in recipients of allogeneic hematopoietic stem cell transplants, in whom it predicted endothelial complications and survival, and it has since shown prognostic value in many other clinical settings [12,13,14]. However, its prognostic significance in IPAH and its relationship with established clinical, functional, and hemodynamic markers have not previously been investigated. We therefore examined whether EASIX carries prognostic information in IPAH.

## 2. Materials and Methods

### 2.1. Study Design and Population

This retrospective observational study was carried out in the cardiology clinic of our hospital, a single center, from December 2021 to March 2026. A total of 297 consecutive stable IPAH outpatients who were admitted to our dedicated IPAH clinic for periodic control visits were enrolled in the study. We did not include patients under 18 years of age, patients whose records lacked the clinical or laboratory values needed to calculate EASIX, or patients without follow-up information. Patients were defined as stable if they had not been hospitalized and had shown no signs of clinical deterioration within the six months before study enrollment. Further exclusion criteria included a lack of data regarding recent hemodynamics, echocardiography, and blood sampling (n = 11); long-term dialysis or other renal replacement therapy (n = 6); active or chronic infection (n = 4); liver or biliary disease or a known primary hematological disorder (n = 1); moderate-to-severe thrombocytopenia (<100 × 10^3^/µL) (n = 2); malignancy (n = 1); chronic inflammatory disease (n = 2); regular treatment with corticosteroids (n = 3), nonsteroidal anti-inflammatory drugs, or anticoagulants (n = 9); previous organ transplantation (n = 1); and any end-stage illness with an expected survival below one year (n = 1). After these 41 exclusions, 256 patients made up the final study population. No platelet-count threshold was applied as an exclusion criterion, so low platelet counts were retained as part of the EASIX measurement. Because patients were enrolled at a routine follow-up visit, most had an established diagnosis (prevalent cohort); the date of the original diagnosis was not systematically recorded, so all analyses were anchored to the baseline (enrollment) visit.

IPAH was diagnosed by right-heart catheterization according to the hemodynamic criteria and contemporary guideline recommendations applicable at the time of each patient’s original diagnosis, after exclusion of identifiable causes of pulmonary hypertension [3,15].

### 2.2. Clinical, Echocardiographic, and Hemodynamic Assessment

We retrieved age, sex, and comorbid conditions, namely hypertension, diabetes mellitus, and coronary artery disease (CAD), from the hospital’s electronic health records. World Health Organization (WHO) functional class was assessed at the clinical examination, and the 6-min walking distance (6-MWD) was measured with the 6-min walk test, in accordance with current guideline recommendations [3].

Right ventricular end-diastolic diameter (RVEDD), tricuspid annular plane systolic excursion (TAPSE), left ventricular ejection fraction (LVEF), and systolic pulmonary artery pressure (sPAP) were obtained from transthoracic echocardiographic assessments performed within the six months prior to study enrollment. Hemodynamic measurements, including mPAP, mean right atrial pressure (mRAP), PVR, and cardiac index (CI) were recorded from the patients’ last right heart catheterization examinations performed within the six months prior to study enrollment. Finger transcutaneous oxygen saturation (SpO_2_), at rest, was recorded from pulse oximetry measurements during physical examination of the patients.

Acute vasoreactivity testing was performed during the diagnostic right-heart catheterization using inhaled nitric oxide (10–20 ppm). A positive acute response was defined as a reduction in mPAP of ≥10 mmHg to reach an absolute value of ≤40 mmHg, with an increased or unchanged cardiac output [3,15].

Medical therapy, including calcium channel blockers (CCB), endothelin receptor antagonists (ERA), phosphodiesterase type 5 inhibitors (PDE5i), prostacyclin analogues, and prostacyclin receptor agonists was recorded as positive if the patients were receiving these medications at enrollment. PAH-specific therapy was categorized as monotherapy or double or triple combination therapy according to the number of PAH-specific drug classes received (ERA, PDE5i, and prostacyclin analogue; CCB were counted separately).

### 2.3. Laboratory Measurements and EASIX Calculation

Routine blood samples were drawn at the enrollment visit, on the same day as the physical examination. Baseline biochemical and hematological parameters, including serum creatinine, LDH, platelet count, serum sodium, albumin, C-reactive protein (CRP), hemoglobin, and N-terminal pro-B-type natriuretic peptide (NT-proBNP), were recorded from the institutional electronic medical records in all patients. The estimated glomerular filtration rate (eGFR) was calculated from the enrollment serum creatinine, age, and sex using the Modification of Diet in Renal Disease (MDRD) formula [16].

EASIX was computed as originally described by Luft et al. [8]:

EASIX = [LDH (U/L) × serum creatinine (mg/dL)]/platelet count (10^9^/L, numerically equal to 10^3^/µL)

### 2.4. Study Endpoint and Follow-Up

The primary endpoint of the study was long-term all-cause mortality. Vital status was ascertained from the hospital’s electronic records and cross-checked against the national civil registry. We measured survival from the baseline evaluation to death; patients still alive were censored at their last documented clinical visit. Because deaths occurring after the last clinical visit were identified through the registry, whereas surviving patients were censored at their last visit, the observed follow-up of survivors may be shorter than that of non-survivors, which may overestimate mortality. Because the date of the registry check was not recorded, surviving patients could not be censored at that date. Follow-up duration is reported as median (IQR).

### 2.5. Statistical Analysis

The primary analyses were run in SPSS Statistics 25.0 (IBM Corp., Armonk, NY, USA); Harrell’s C-index, likelihood-ratio and DeLong tests, Schoenfeld residual tests and bootstrap analyses were performed in Python 3.13 (NumPy 2.5 and SciPy 1.18) with custom code, which reproduced the SPSS Cox regression estimates. We checked the normality of continuous data with the Kolmogorov–Smirnov test. Continuous data are given as mean ± standard deviation (SD) when normally distributed and as median (interquartile range [IQR]) otherwise; categorical data are given as counts and percentages. For two-group comparisons we used Student’s *t*-test for independent samples or the Mann–Whitney U test for continuous data and the chi-square or Fisher’s exact test for categorical data. Given the right-skewed distribution of NT-proBNP values, a base-10 logarithmic transformation was applied prior to statistical analysis, and the transformed variable was expressed as logNT-proBNP; HRs for logNT-proBNP therefore refer to a 10-fold increase in NT-proBNP. EASIX was also right-skewed and is described by its median, IQR, and range, together with its mean ± SD.

We used receiver operating characteristic (ROC) curves to assess how well EASIX separated survivors from non-survivors and the Youden index to choose the optimal EASIX cut-off. Survival analysis was performed using the Kaplan–Meier method and compared using the log-rank test. The discriminative ability of EASIX, mPAP, and logNT-proBNP to predict mortality was evaluated using ROC curve analysis, with the area under the curve (AUC) calculated for each parameter. Associations with mortality were examined with Cox proportional hazards regression. Because only 41 deaths occurred, the multivariable models were restricted to six predictors (approximately seven events per variable): EASIX, age, sex, and the three non-invasive variables of the ESC/ERS four-strata follow-up risk assessment, namely WHO functional class (III–IV vs. I–II), 6-MWD, and logNT-proBNP [3]. LDH, creatinine, and platelet count make up EASIX, and eGFR is derived from creatinine, so these variables were not entered as separate covariates. Model 1 treated EASIX as continuous (hazard per 1-unit increase), and Model 2 dichotomized it at the ROC-derived cut-off. Multicollinearity was assessed with variance inflation factors (VIFs). The proportional hazards assumption was tested with scaled Schoenfeld residuals, for each covariate and globally, and by inspection of log-minus-log survival plots; when a covariate did not satisfy the assumption, the models were refitted stratified by that covariate. Further sensitivity analyses expressed EASIX per SD increase and per doubling (log2-transformed EASIX). The incremental prognostic value of EASIX was evaluated by comparing a base model (WHO-FC, 6-MWD, and logNT-proBNP) with the same model plus EASIX, using Harrell’s C-index (95% CI from 1000 bootstrap samples) and a likelihood-ratio test. The AUCs of EASIX, mPAP, and logNT-proBNP were compared with the DeLong test. To assess the stability of the data-derived cut-off, the Youden-optimal cut-off was re-estimated in 1000 bootstrap samples, giving a bootstrap 95% CI for the cut-off and optimism-corrected estimates of its sensitivity and specificity. Effect sizes are expressed as hazard ratios (HRs) with 95% confidence intervals (CIs), and statistical significance was set at a two-sided *p* < 0.05.

Generative artificial intelligence (Claude, Claude Opus 5.5, Anthropic, PBC, San Francisco, USA) was not used to design the study, to collect the data, or to interpret the results. It was used to help write the Python code for the Harrell’s C-index, likelihood-ratio, DeLong, Schoenfeld residual, and bootstrap analyses and the code that generated the figures; this code was reviewed by the authors. Its use in drafting the text and in applying the journal format is described in the Acknowledgments.

## 3. Results

**Figure 1:**
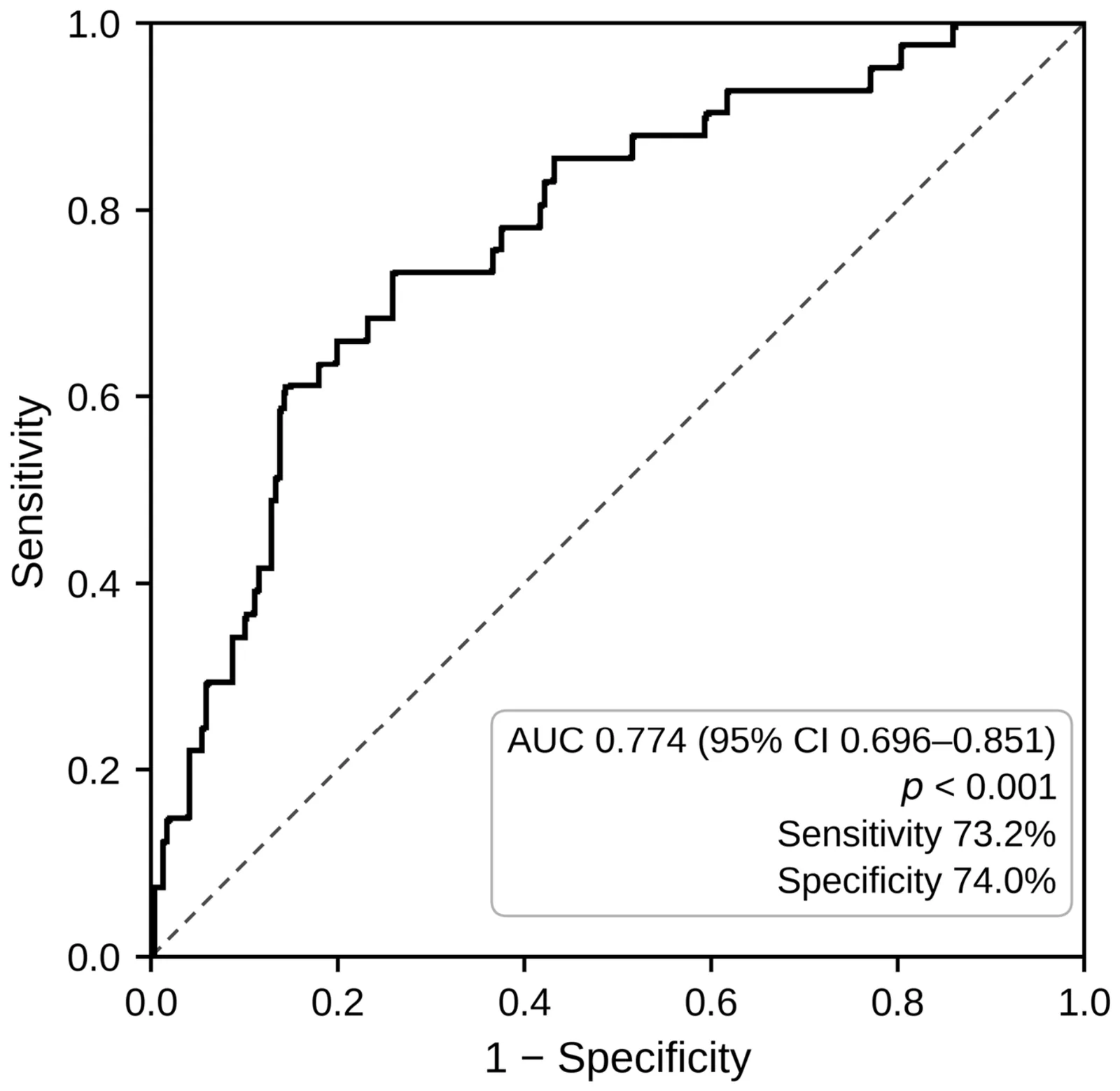
Receiver operating characteristic curve analysis for the optimal cut-off value of EASIX in predicting all-cause mortality. The diagonal reference line indicates no discrimination. EASIX, endothelial activation, and stress index; AUC, area under the receiver operating characteristic curve; CI, confidence interval.

**Figure 2:**
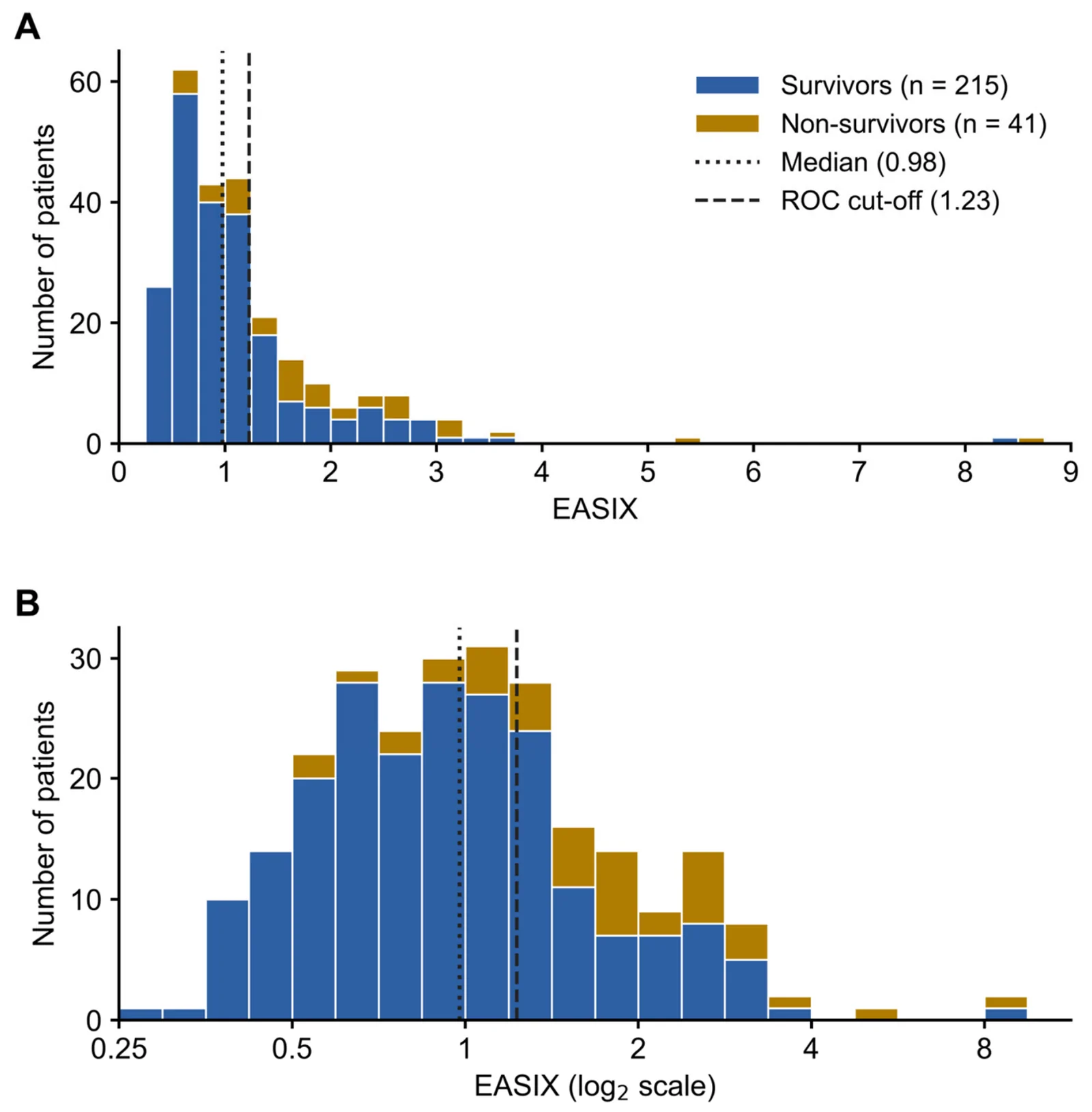
Distribution of EASIX in the study cohort (n = 256), overall and by vital status. (**A**) Histogram of EASIX on the original scale. (**B**) Histogram of EASIX on a log_2_ scale. Bars are stacked by vital status (survivors, n = 215; non-survivors, n = 41). EASIX, endothelial activation and stress index; ROC, receiver operating characteristic.

The final analysis included 256 patients with IPAH. During a median follow-up of 35 (IQR 21–46) months, all-cause mortality occurred in 41 patients (16.0%). Based on the optimal ROC-derived cut-off value of EASIX (Figure 1), the study population was divided into a low-EASIX group (EASIX < 1.23, n = 170) and a high-EASIX group (EASIX ≥ 1.23, n = 86). EASIX was right-skewed, with a median of 0.98 (IQR 0.65–1.46; range 0.29–8.64) and a mean ± SD of 1.24 ± 0.98 in the whole cohort (Figure 2).

Baseline demographic and clinical characteristics according to EASIX groups are presented in Table 1. Age, sex, and the prevalence of diabetes mellitus, hypertension, and CAD were similar between the two groups. Patients in the high-EASIX group had significantly lower serum albumin levels [39.0 (35.7–41.0) vs. 40.0 (38.42–43.9) g/L; *p* = 0.007], eGFR (45.48 ± 15.85 vs. 67.48 ± 21.62 mL/min/1.73 m^2^; *p* < 0.001), platelet count (194.61 ± 70.21 vs. 252.08 ± 63.26 × 10^3^/µL; *p* < 0.001), and serum sodium levels (133.97 ± 3.13 vs. 135.29 ± 3.60 mmol/L; *p* = 0.004) than those in the low-EASIX group. In contrast, serum creatinine (1.47 ± 0.45 vs. 1.02 ± 0.28 mg/dL; *p* < 0.001), serum LDH (264.32 ± 66.81 vs. 185.68 ± 46.57 U/L; *p* < 0.001), and logNT-proBNP [3.14 (2.91–3.25) vs. 2.97 (2.80–3.16); *p* < 0.001] were significantly elevated in patients with high EASIX. CRP was also numerically higher in this group, but the difference was not significant (*p* = 0.078). Hemoglobin levels were comparable between the groups. Use of ERA, PDE5i, prostacyclin analogues, prostacyclin receptor agonists, and CCB was similar in both groups. Overall, 150 patients (58.6%) received PAH-specific monotherapy, 84 (32.8%) double, and 16 (6.3%) triple combination therapy, and 6 (2.3%) received CCB alone; this distribution did not differ significantly between the EASIX groups (*p* = 0.126).

The echocardiographic and right heart catheterization features are summarized in Table 2. Patients with EASIX ≥ 1.23 had a larger RVEDD [31.0 (29.0–35.0) vs. 30.0 (28.0–32.0) mm; *p* = 0.004], higher sPAP (69.65 ± 20.28 vs. 62.28 ± 20.26 mmHg; *p* = 0.006), and lower TAPSE (13.88 ± 2.70 vs. 15.28 ± 3.02 mm; *p* < 0.001) than patients with EASIX < 1.23. LVEF was similar between the two groups (*p* = 0.218). Hemodynamic assessment demonstrated that the high-EASIX group had higher mPAP (34.34 ± 6.71 vs. 31.54 ± 5.99 mmHg; *p* = 0.001), mRAP [7.0 (6.0–9.0) vs. 6.0 (5.0–8.0) mmHg; *p* = 0.003], and PVR (5.20 ± 2.19 vs. 4.57 ± 2.33 WU; *p* = 0.040). Conversely, CI was significantly lower in patients with high EASIX (2.57 ± 0.68 vs. 2.88 ± 0.75 L/min/m^2^; *p* = 0.002). Vasoreactivity test positivity was observed in 43 patients (16.8%) overall and was similar between the low- and high-EASIX groups (29 [17.1%] vs. 14 [16.3%], respectively; *p* = 0.875). Patients in the high-EASIX group also had lower finger SpO_2_ [91.0 (89.0–93.0) vs. 93.0 (91.0–94.0)%; *p* < 0.001] and shorter 6-MWD (206.78 ± 81.89 vs. 253.88 ± 80.55 m; *p* < 0.001). WHO functional class III or IV was more frequent in the high-EASIX group (62.8% vs. 33.5%; *p* < 0.001). All-cause mortality was markedly higher in the high-EASIX group than in the low-EASIX group (34.9% vs. 6.5%; *p* < 0.001).

**Figure 3:**
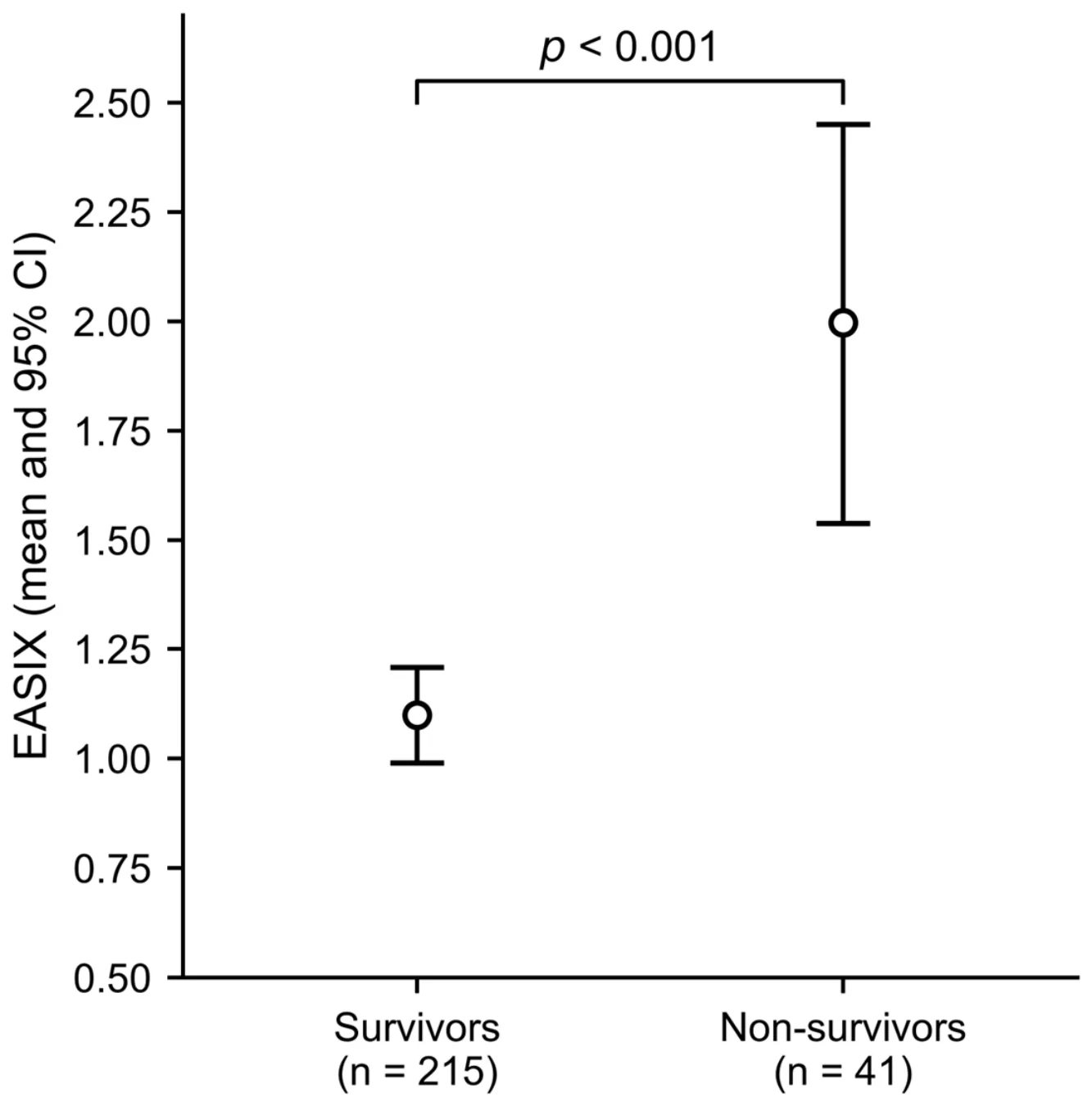
Error plot comparing EASIX between survivors (n = 215) and non-survivors (n = 41). Circles mark mean values, with 95% confidence intervals shown as bars. EASIX, endothelial activation, and stress index.

EASIX showed significant discrimination for long-term all-cause mortality on ROC analysis (AUC 0.774, 95% CI 0.696–0.851; *p* < 0.001). An EASIX value of 1.23 was identified as the optimal cut-off, providing 73.2% sensitivity and 74.0% specificity for all-cause mortality (Figure 1). In 1000 bootstrap samples, the 95% CI of the optimal cut-off was 0.97–1.64, and the optimism-corrected sensitivity and specificity were 69.4% and 73.0%, respectively. Non-survivors had significantly higher EASIX values than survivors (median 1.68 [IQR 1.07–2.48] vs. 0.91 [IQR 0.64–1.25]; *p* < 0.001; error plot in Figure 3).

Table 3 presents the univariable and multivariable Cox proportional hazards regression analyses of predictors of all-cause mortality, with EASIX evaluated as both a continuous variable (Model 1) and a categorical variable using the ROC-derived cut-off of 1.23 (Model 2). In the univariable analyses, continuous EASIX was associated with all-cause mortality (HR 1.440, 95% CI 1.249–1.661; *p* < 0.001). When EASIX was categorized, patients with EASIX ≥ 1.23 had approximately six times the hazard of all-cause mortality compared with those below the cut-off (HR 6.057, 95% CI 3.033–12.096; *p* < 0.001). WHO-FC III–IV and logNT-proBNP were also positively associated with mortality, whereas higher 6-MWD was associated with a lower hazard of death in the univariable analyses. After multivariable adjustment, continuous EASIX remained independently associated with all-cause mortality in Model 1 (HR 1.528, 95% CI 1.255–1.860; *p* < 0.001). WHO-FC III–IV (HR 4.674, 95% CI 1.819–12.011; *p* = 0.001) and logNT-proBNP (HR 24.321, 95% CI 5.107–115.836; *p* < 0.001) were also independently associated with mortality. In contrast, 6-MWD was not significantly associated with mortality after adjustment (HR 1.000, 95% CI 0.996–1.005; *p* = 0.851). In Model 2, EASIX ≥ 1.23 remained independently associated with all-cause mortality, with an approximately fourfold higher hazard compared with EASIX < 1.23 (HR 3.989, 95% CI 1.962–8.111; *p* < 0.001). WHO-FC III–IV (HR 4.431, 95% CI 1.725–11.386; *p* = 0.002) and logNT-proBNP (HR 8.482, 95% CI 1.953–36.835; *p* = 0.004) also remained independent predictors of mortality, whereas 6-MWD was not statistically significant (HR 0.999, 95% CI 0.995–1.004; *p* = 0.800). The VIF values were low in both models (Model 1: 1.100–1.592; Model 2: 1.120–1.353), indicating no evidence of substantial multicollinearity among the predictors included in the respective multivariable models. The proportional hazards assumption was not rejected for EASIX (Schoenfeld test *p* = 0.18 in Model 1 and *p* = 0.59 in Model 2) and WHO-FC or logNT-proBNP (all *p* > 0.05), but it was not met for 6-MWD (*p* = 0.015 and *p* = 0.008, respectively), giving global Schoenfeld test *p* values of 0.04 for Model 1 and 0.02 for Model 2. When the models were stratified by 6-MWD (below vs. at or above the median), the HR for EASIX was 1.519 (95% CI 1.252–1.842) in Model 1 and 4.086 (95% CI 2.002–8.338) in Model 2.

In sensitivity analyses, adding age and sex to the multivariable models gave an HR for EASIX of 1.496 (95% CI 1.221–1.833) in Model 1 and 3.853 (95% CI 1.884–7.881) in Model 2, within 4% of the main estimates. Expressed per SD increase (1 SD = 0.98) and per doubling, the adjusted HR for EASIX was 1.517 (95% CI 1.250–1.841) and 2.294 (95% CI 1.579–3.332), respectively. The base model, including WHO-FC, 6-MWD, and logNT-proBNP had a Harrell’s C-index of 0.784 (95% CI 0.722–0.866). Adding continuous EASIX changed the C-index to 0.812 (95% CI 0.747–0.896; likelihood-ratio test *p* < 0.001).

**Figure 4:**
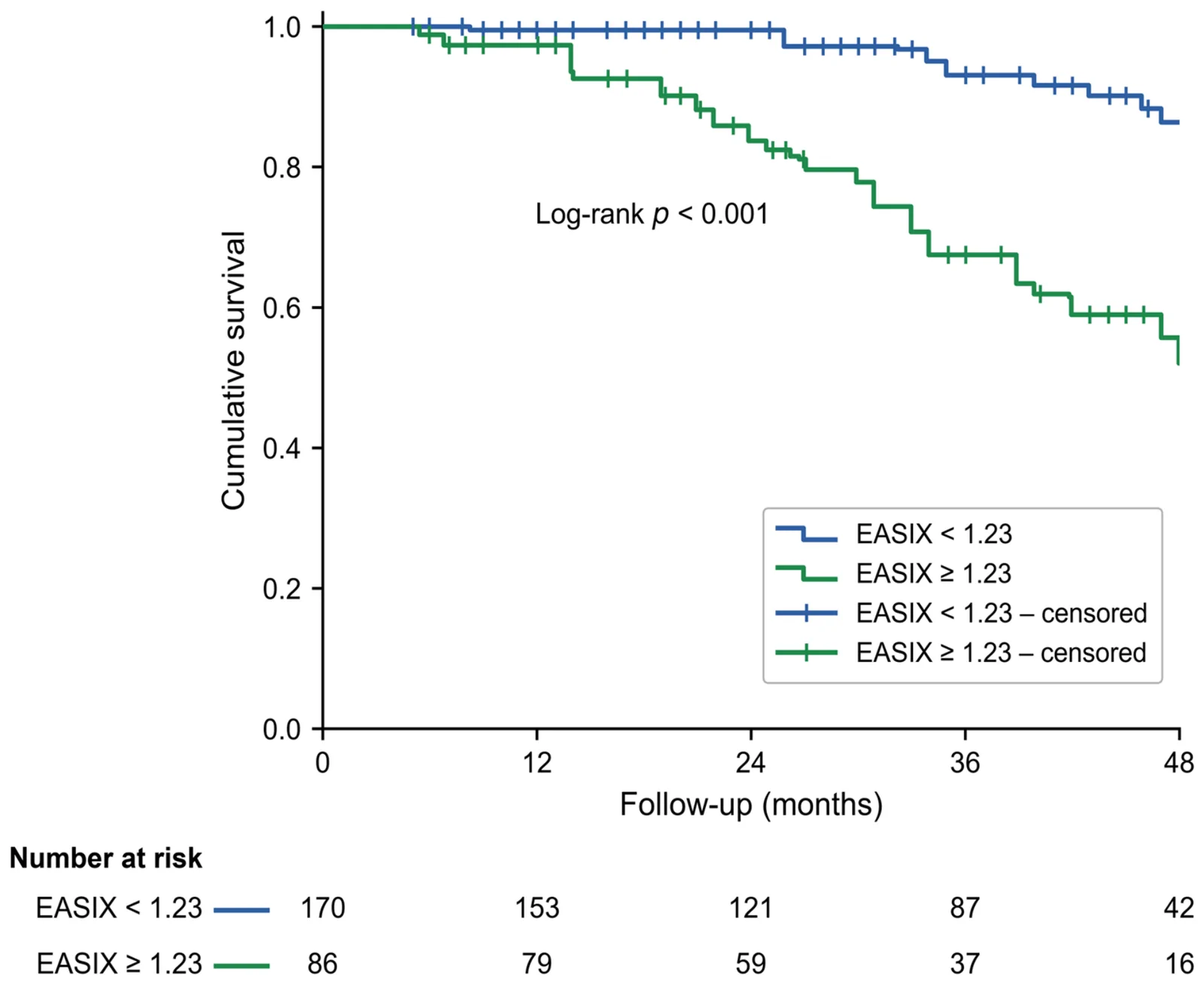
Kaplan–Meier analysis of cumulative survival stratified by EASIX < 1.23 and EASIX ≥ 1.23. The number of patients at risk in each group is shown below the plot. EASIX, endothelial activation, and stress index.

The Kaplan–Meier curves separated clearly when patients were grouped by the EASIX cut-off of 1.23. Patients with EASIX ≥ 1.23 exhibited significantly lower cumulative survival than those with EASIX < 1.23 during follow-up (log-rank *p* < 0.001; Figure 4).

**Figure 5:**
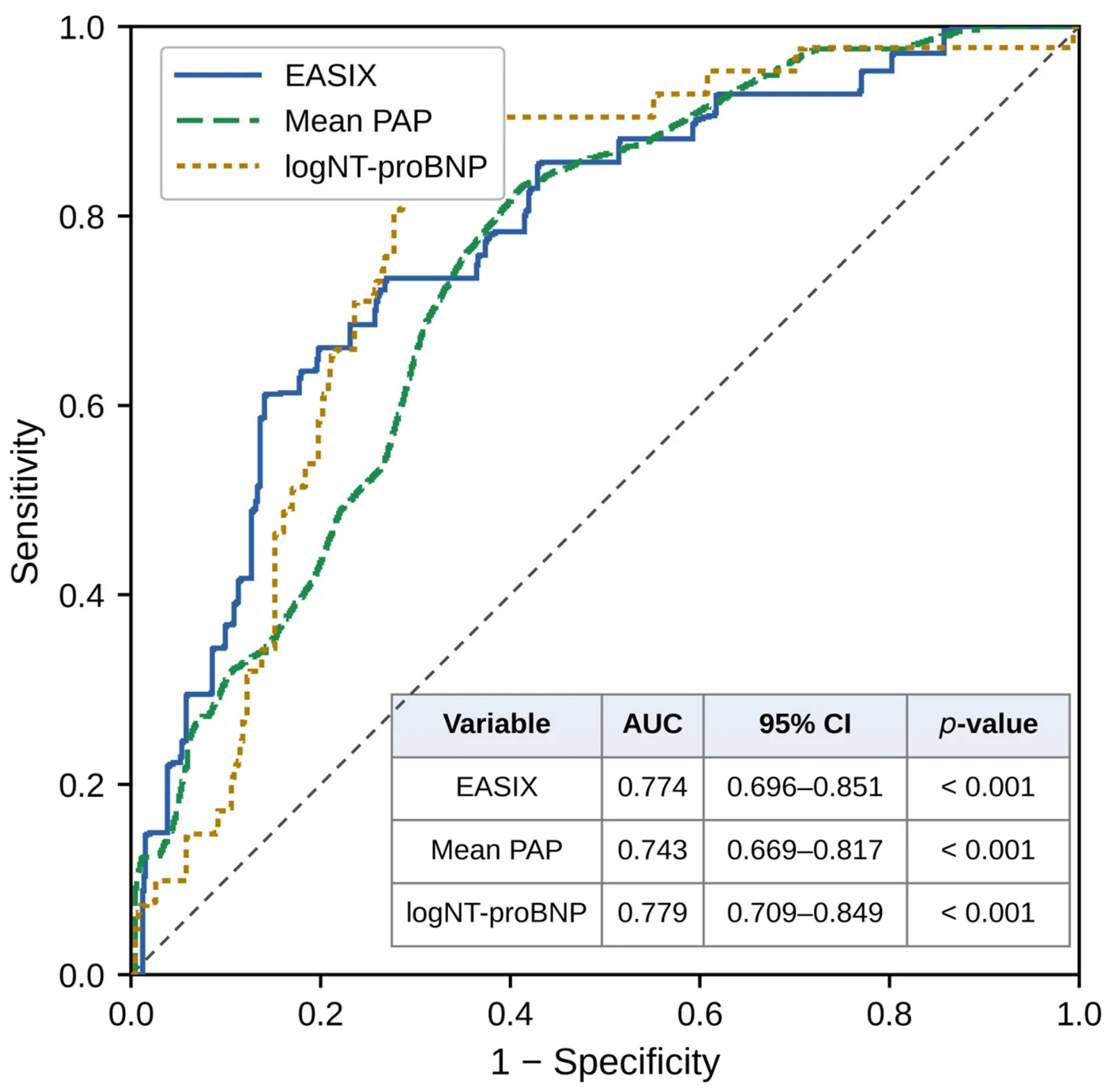
Receiver operating characteristic analysis comparing EASIX, mean PAP, and logNT-proBNP as predictors of all-cause mortality. The inset table reports the AUC with its 95% confidence interval for each variable; the diagonal reference line indicates no discrimination. EASIX, endothelial activation and stress index; AUC, area under the receiver operating characteristic curve; CI, confidence interval; NT-proBNP, N-terminal pro-B-type natriuretic peptide; PAP, pulmonary artery pressure.

Figure 5 compares the ROC curves of EASIX, mPAP, and logNT-proBNP for all-cause mortality. The AUC was 0.774 for EASIX (95% CI 0.696–0.851; *p* < 0.001), 0.743 for mPAP (95% CI 0.669–0.817; *p* < 0.001), and 0.779 for logNT-proBNP (95% CI 0.709–0.849; *p* < 0.001). On DeLong testing, the AUC of EASIX did not differ significantly from that of logNT-proBNP (*p* = 0.890) or mPAP (*p* = 0.470).

## 4. Discussion

We investigated the prognostic value of EASIX in stable outpatients with IPAH. In our study, higher EASIX values were independently associated with an increased risk of all-cause mortality in patients with IPAH. Furthermore, patients with higher EASIX values showed poorer functional capacity and worse hemodynamic characteristics. We are not aware of any earlier study that has examined EASIX, which combines serum LDH, serum creatinine, and platelet count, in IPAH.

Current guideline-directed risk assessment in IPAH incorporates functional capacity, natriuretic peptides, imaging findings, and hemodynamic parameters; however, routinely used circulating biomarkers predominantly reflect myocardial wall stress [3]. The association between various inflammatory biomarkers and IPAH has been increasingly recognized following the identification of endothelial dysfunction and inflammation as key pathophysiological mechanisms underlying the disease. CRP, serum albumin, the neutrophil-to-lymphocyte ratio, the systemic immune-inflammation index, and the systemic inflammatory response index have each been linked to the development and prognosis of IPAH [17,18,19,20,21,22]. EASIX, as a relatively novel marker of endothelial activation and stress, has emerged as a promising prognostic marker after initially being introduced as a predictor of adverse outcomes in patients undergoing allogeneic hematopoietic stem cell transplantation [8]. Subsequently, the clinical importance of EASIX has been demonstrated across various cardiovascular settings, including CAD, HF, acute myocardial infarction (AMI), and patients undergoing transcatheter aortic valve replacement (TAVR) [12,13,23,24,25]. Finke et al. demonstrated the prognostic value of EASIX in patients with CAD and suggested it as a potential biomarker for predicting mortality, irrespective of whether it was assessed before or after cardiac catheterization [13]. In acute decompensated HF, Özlek et al. found that patients with a higher EASIX on admission were significantly more likely to die in hospital [14]. Mousa Basha et al. aimed to assess the relationship between EASIX and all-cause mortality in patients undergoing TAVR. In their study, patients with an EASIX ≥ 1.65 had significantly higher 3-year all-cause mortality compared with those with lower EASIX (45.8% vs. 27.7%; *p* < 0.001), and EASIX was found to be a significant predictor of 3-year all-cause mortality [24]. They concluded that integrating EASIX with traditional cardiac biomarkers may enhance risk stratification in TAVR patients and improve personalized care. In a retrospective cohort study of 1036 patients with AMI, higher EASIX scores were associated with increased 30-day mortality, suggesting EASIX as a valuable prognostic tool that may inform clinical management strategies to improve outcomes in AMI [25]. Across these conditions, EASIX may reflect a composite of cellular injury, impaired tissue perfusion, renal dysfunction, and systemic stress rather than endothelial activation alone. In IPAH, higher EASIX may partly reflect the consequences of more advanced right-heart failure, including reduced cardiac output, venous congestion, and cardiorenal dysfunction, while LDH may reflect tissue hypoxia or cellular injury, and platelet count may also be influenced by disease-related and treatment-related factors, including prostacyclin therapy. Therefore, the association between EASIX and mortality observed in our cohort should not be interpreted as evidence that endothelial activation per se is the principal mechanism underlying its prognostic value [9,10,11]. Considered together, these parameters may also reflect the broader systemic impact of advanced pulmonary vascular disease more comprehensively than any single biomarker. Previous pulmonary studies have evaluated EASIX in acute pulmonary embolism, acute respiratory distress syndrome, and critically ill patients with chronic obstructive pulmonary disease; however, its relationship with invasive hemodynamics, disease severity, and survival in IPAH has not previously been examined [26,27,28].

Higher EASIX was consistently associated with a more advanced IPAH phenotype. Patients with EASIX ≥ 1.23 had shorter 6-MWD, lower finger SpO_2_, and worse functional capacity. They also had larger right ventricular dimensions, lower TAPSE, higher mPAP and mRAP, increased PVR, and lower CI. These findings suggest that EASIX is not simply an isolated laboratory marker of mortality but may identify patients with more advanced pulmonary vascular disease and impaired right ventricular adaptation. The absence of a significant difference in LVEF further suggests that this adverse profile was predominantly related to pulmonary vascular and right-sided cardiac involvement.

The association between EASIX and mortality was observed both when EASIX was analyzed as a continuous variable and when it was dichotomized. Each one-unit increase in EASIX was independently associated with a 52.8% higher hazard of death, and EASIX ≥ 1.23 with an approximately fourfold higher hazard. Because both models use the same variable, their agreement is not an independent confirmation; it shows only that the association does not depend on dichotomization at a single data-derived threshold, which itself has not been externally validated. The continuous model suggests that risk rises progressively as EASIX increases, and this was also seen when EASIX was expressed per SD increase or per doubling. The association persisted after adjustment for WHO functional class, 6-MWD, and logNT-proBNP—the non-invasive variables of the ESC/ERS four-strata risk model—but residual confounding by aspects of disease severity not captured by these variables cannot be excluded.

For mortality, EASIX reached an AUC of 0.774, with balanced sensitivity and specificity. Its AUC was numerically similar to that of logNT-proBNP and higher than that of mPAP, but neither difference was statistically significant on DeLong testing. Adding EASIX to a base model with WHO-FC, 6-MWD, and logNT-proBNP changed Harrell’s C-index from 0.784 to 0.812 (likelihood-ratio test *p* < 0.001). These analyses describe an association with mortality and a change in model discrimination within a single cohort; they do not establish clinical utility or improved risk stratification. Whether EASIX adds information to complete validated risk tools, such as the ESC/ERS four-strata model, COMPERA 2.0, or REVEAL Lite 2, must be tested in prospective multicenter studies [3,4].

### Study Limitations

Our study has several limitations. First, its retrospective single-center design and the inclusion of predominantly prevalent IPAH patients introduce potential selection and survivor bias and may limit generalizability [29]. The date of the original diagnosis was not systematically recorded, so the time from diagnosis to enrollment could not be reported or adjusted for; as in other prevalent PAH cohorts, survival was therefore analyzed from a standardized study-entry visit [29]. Second, the EASIX cut-off of 1.23 was derived and evaluated in the same cohort. Bootstrap resampling only partly corrects for the resulting optimism, and conventional ROC analysis does not account for censoring; the cut-off should therefore be regarded as exploratory, and the reported discriminatory performance requires external validation. Third, with 41 deaths the multivariable models could include only a few covariates, so residual confounding by hemodynamic, imaging, or other variables cannot be excluded, and the proportional hazards assumption was not met for 6-MWD; EASIX itself met the assumption, and stratification by 6-MWD did not materially change its estimate, but the power to detect departures from proportional hazards was limited. Fourth, deaths after the last clinical visit were captured from the national registry, whereas surviving patients were censored at their last visit. This asymmetry shortens the observed follow-up of survivors and may overestimate mortality. Because the date of the registry check was not recorded, survivors could not be censored at that date, and the size of this bias could not be quantified. Changes in PAH-specific therapy during follow-up and possible effects of prostacyclin therapy on platelet count may have influenced EASIX and outcomes. Sixth, specific endothelial or inflammatory biomarkers were not measured, and the observed association may therefore reflect the systemic consequences of right-heart failure rather than endothelial activation per se. Finally, cause-of-death data were not reliably available, and the incremental prognostic value of EASIX was assessed only against WHO-FC, 6-MWD, and NT-proBNP, not against complete validated PAH risk scores.

## 5. Conclusions

In this single-center cohort of patients with IPAH, higher EASIX was associated with a more advanced clinical, functional, echocardiographic, and hemodynamic disease profile and was independently associated with long-term all-cause mortality, both as a continuous variable and when dichotomized at a data-derived cut-off. These findings describe an association and do not establish clinical utility. Whether EASIX adds prognostic information to contemporary multiparametric risk-stratification tools and whether the 1.23 threshold is reproducible must be tested in prospective multicenter cohorts.

## Figures and Tables

**Table 1:** Baseline demographic and laboratory characteristics of the study patients according to the EASIX group.

Variables	All (n = 256)	EASIX < 1.23 (n = 170)	EASIX ≥ 1.23 (n = 86)	*p*-Value
Age, years	53.34 ± 11.01	54.00 ± 10.91	52.05 ± 11.14	0.180
Sex (female), n (%)	178 (69.5)	118 (69.4)	60 (69.8)	0.953
Diabetes mellitus, n (%)	10 (3.9)	5 (2.9)	5 (5.8)	0.262
Hypertension, n (%)	19 (7.4)	14 (8.2)	5 (5.8)	0.485
CAD, n (%)	14 (5.5)	8 (4.7)	6 (7.0)	0.450
**Laboratory findings**				
CRP, mg/L	1.96 (0.99–4.95)	1.55 (0.99–3.52)	2.80 (0.97–6.58)	0.078
Albumin, g/L	40.0 (37.0–41.8)	40.0 (38.42–43.9)	39.0 (35.7–41.0)	0.007
Serum creatinine, mg/dL	1.17 ± 0.40	1.02 ± 0.28	1.47 ± 0.45	<0.001
eGFR, mL/min/1.73 m^2^	60.09 ± 22.40	67.48 ± 21.62	45.48 ± 15.85	<0.001
LDH, U/L	212.10 ± 65.65	185.68 ± 46.57	264.32 ± 66.81	<0.001
Platelet count, ×10^3^/µL	232.77 ± 70.96	252.08 ± 63.26	194.61 ± 70.21	<0.001
Hemoglobin, g/dL	11.36 ± 1.47	11.44 ± 1.43	11.21 ± 1.55	0.420
Serum sodium, mmol/L	134.85 ± 3.50	135.29 ± 3.60	133.97 ± 3.13	0.004
logNT-proBNP	3.02 (2.82–3.21)	2.97 (2.80–3.16)	3.14 (2.91–3.25)	<0.001
EASIX, median (IQR)	0.98 (0.65–1.46)	0.74 (0.58–0.97)	1.86 (1.44–2.54)	–
EASIX, mean ± SD	1.24 ± 0.98	0.77 ± 0.25	2.17 ± 1.22	–
**PAH-specific therapy, n (%)**				
ERA	210 (82.0)	139 (81.8)	71 (82.6)	0.876
PDE5i	80 (31.3)	48 (28.2)	32 (37.2)	0.143
Prostacyclin analogues	76 (29.7)	45 (26.5)	31 (36.0)	0.113
CCB	50 (19.5)	34 (20.0)	16 (18.6)	0.790
Prostacyclin receptor agonists	19 (7.4)	14 (8.2)	5 (5.8)	0.485
**Combinations of PAH-specific therapy, n (%)**				0.126
Monotherapy	150 (58.6)	107 (62.9)	43 (50.0)	
Double combination	84 (32.8)	49 (28.8)	35 (40.7)	
Triple combination	16 (6.3)	9 (5.3)	7 (8.1)	
CCB only	6 (2.3)	5 (2.9)	1 (1.2)	

Continuous variables are presented as mean ± standard deviation or median (interquartile range); categorical variables are presented as n (%). CAD, coronary artery disease; CCB, calcium channel blocker; CRP, C-reactive protein; EASIX, endothelial activation and stress index; eGFR, estimated glomerular filtration rate; ERA, endothelin receptor antagonist; LDH, lactate dehydrogenase; NT-proBNP, N-terminal pro-B-type natriuretic peptide; PAH, pulmonary arterial hypertension; PDE5i, phosphodiesterase type 5 inhibitor. Platelet count in ×10^3^/µL (equivalent to ×10^9^/L). PAH-specific therapy counts ERA, PDE5i, and prostacyclin analogues; CCBs are not counted. The *p*-value for PAH-specific therapy refers to the overall distribution (Fisher–Freeman–Halton exact test).

**Table 2:** Echocardiographic, hemodynamic, and clinical features of the study patients according to the EASIX group.

Variables	All (n = 256)	EASIX < 1.23 (n = 170)	EASIX ≥ 1.23 (n = 86)	*p*-Value
RVEDD, mm	30.0 (28.0–33.0)	30.0 (28.0–32.0)	31.0 (29.0–35.0)	0.004
sPAP, mmHg	64.75 ± 20.53	62.28 ± 20.26	69.65 ± 20.28	0.006
TAPSE, mm	14.81 ± 2.98	15.28 ± 3.02	13.88 ± 2.70	<0.001
LVEF, %	62.33 ± 2.59	62.48 ± 2.56	62.05 ± 2.65	0.218
Mean PAP, mmHg	32.48 ± 6.37	31.54 ± 5.99	34.34 ± 6.71	0.001
Mean RAP, mmHg	7.0 (5.0–8.0)	6.0 (5.0–8.0)	7.0 (6.0–9.0)	0.003
PVR, WU	4.79 ± 2.30	4.57 ± 2.33	5.20 ± 2.19	0.040
Vasoreactivity test positivity	43 (16.8%)	29 (17.1%)	14 (16.3%)	0.875
CI, L/min/m^2^	2.78 ± 0.74	2.88 ± 0.75	2.57 ± 0.68	0.002
Finger SpO_2_, %	92.0 (90.0–93.0)	93.0 (91.0–94.0)	91.0 (89.0–93.0)	<0.001
6-MWD, m	238.05 ± 83.86	253.88 ± 80.55	206.78 ± 81.89	<0.001
WHO-FC, n (%)				<0.001
I	29 (11.3)	27 (15.9)	2 (2.3)	
II	116 (45.3)	86 (50.6)	30 (34.9)	
III	91 (35.5)	50 (29.4)	41 (47.7)	
IV	20 (7.8)	7 (4.1)	13 (15.1)	
All-cause mortality, n (%)	41 (16.0)	11 (6.5)	30 (34.9)	<0.001

Continuous variables are presented as mean ± standard deviation or median (interquartile range); categorical variables are presented as n (%). 6-MWD, 6-min walking distance; CI, cardiac index; EASIX, endothelial activation and stress index; LVEF, left ventricular ejection fraction; PAP, pulmonary artery pressure; PVR, pulmonary vascular resistance; RAP, right atrial pressure; RVEDD, right ventricular end-diastolic diameter; SpO_2_, peripheral oxygen saturation; sPAP, systolic pulmonary artery pressure; TAPSE, tricuspid annular plane systolic excursion; WHO-FC, World Health Organization functional class; WU, Wood units.

**Table 3:** Univariable and multivariable Cox proportional hazards regression analyses for predictors of all-cause mortality.

Model 1. EASIX as a Continuous Variable					
Variable	Univariable Analysis		Multivariable Analysis		VIF
	HR (95% CI)	*p*-Value	HR (95% CI)	*p*-Value	
EASIX (per 1-unit increase)	1.440 (1.249–1.661)	<0.001	1.528 (1.255–1.860)	<0.001	1.159
Age, years	1.010 (0.980–1.042)	0.523	-	-	-
Male gender	1.227 (0.601–2.504)	0.574	-	-	-
WHO-FC III–IV	7.947 (3.342–18.900)	<0.001	4.674 (1.819–12.011)	0.001	1.348
6-MWD, m	0.994 (0.990–0.997)	0.001	1.000 (0.996–1.005)	0.851	1.592
logNT-proBNP	27.875 (6.373–121.930)	<0.001	24.321 (5.107–115.836)	<0.001	1.100
**Model 2. EASIX Categorized According to the ROC-Derived Cut-Off Value**					
**Variable**	**Univariable Analysis**		**Multivariable Analysis**		**VIF**
	**HR (95% CI)**	***p*** **-Value**	**HR (95% CI)**	***p*** **-Value**	
EASIX ≥ 1.23	6.057 (3.033–12.096)	<0.001	3.989 (1.962–8.111)	<0.001	1.138
Age, years	1.010 (0.980–1.042)	0.523	-	-	-
Male gender	1.227 (0.601–2.504)	0.574	-	-	-
WHO-FC III–IV	7.947 (3.342–18.900)	<0.001	4.431 (1.725–11.386)	0.002	1.333
6-MWD, m	0.994 (0.990–0.997)	0.001	0.999 (0.995–1.004)	0.800	1.353
logNT-proBNP	27.875 (6.373–121.930)	<0.001	8.482 (1.953–36.835)	0.004	1.120

Values are hazard ratios (HRs) with 95% confidence intervals (CIs). 6-MWD, 6-min walking distance; CI, confidence interval; EASIX, endothelial activation and stress index; HR, hazard ratio; NT-proBNP, N-terminal pro-B-type natriuretic peptide; VIF, variance inflation factor; WHO-FC, World Health Organization functional class.

## Data Availability

The dataset can be obtained from the corresponding author on reasonable request. It is not publicly available because of institutional privacy and ethical restrictions.

## Abbreviations

The following abbreviations are used in this manuscript:

**Abbreviation**	**Definition**
6-MWD	6-min walking distance
AMI	Acute myocardial infarction
AUC	Area under the curve
CAD	Coronary artery disease
CCB	Calcium channel blocker
CI	Cardiac index
CRP	C-reactive protein
EASIX	Endothelial activation and stress index
eGFR	Estimated glomerular filtration rate
ERA	Endothelin receptor antagonist
HF	Heart failure
HR	Hazard ratio
IPAH	Idiopathic pulmonary arterial hypertension
IQR	Interquartile range
LDH	Lactate dehydrogenase
LVEF	Left ventricular ejection fraction
mPAP	Mean pulmonary artery pressure
mRAP	Mean right atrial pressure
NT-proBNP	N-terminal pro-B-type natriuretic peptide
PAH	Pulmonary arterial hypertension
PDE5i	Phosphodiesterase type 5 inhibitor
PVR	Pulmonary vascular resistance
ROC	Receiver operating characteristic
RVEDD	Right ventricular end-diastolic diameter
SD	Standard deviation
SpO_2_	Peripheral oxygen saturation
sPAP	Systolic pulmonary artery pressure
TAPSE	Tricuspid annular plane systolic excursion
TAVR	Transcatheter aortic valve replacement
VIF	Variance inflation factor
WHO	World Health Organization
WU	Wood units
